# Two isomers of [1-benzyl-4-(pyridin-2-yl-κ*N*)-1*H*-1,2,3-triazole-κ*N* ^3^]di­chlorido­bis­(dimethyl sulfoxide-κ*S*)ruthenium(II)

**DOI:** 10.1107/S2056989019008375

**Published:** 2019-07-04

**Authors:** Fatemeh Khamespanah, Andrew W. Maverick

**Affiliations:** a Department of Chemistry, Louisiana State University, Baton Rouge, Louisiana, 70803, USA

**Keywords:** crystal structure, ruthenium(II) complex, pyridyl­triazole

## Abstract

The structures of two isomers of the title compound, [RuCl_2_(C_14_H_12_N_4_)(C_2_H_6_OS)_2_], **2** and **3**, are reported. Isomers **2** and **3** are produced by reaction of the pyridyl­triazole ligand 1-benzyl-4-(pyridin-2-yl)-1*H*-1,2,3-triazole (bpt) (**1**) with *fac*-[RuCl_2_(DMSO-*S*)_3_(DMSO-*O*)]. Reaction in acetone produces *ca* 95% **2**, which is the *OC*-6-14 isomer, with *cis* DMSO and *trans* chlorido ligands, and 5% **3** (the *OC*-6-32 isomer, with *cis* DMSO and *cis* chlorido ligands, and the pyridyl moiety of bpt *trans* to DMSO). Reaction in refluxing toluene initially forms **2**, which slowly isomerizes to **3**.

## Abstract 2

Reaction of [RuCl_2_(DMSO)_4_] with 1-benzyl-4-(pyridin-2-yl)-1*H*-1,2,3-triazole yields two of the four possible isomers of the title compound.

## Chemical context   

Many 1,2,3-triazole-based ligands have been prepared by copper(I) catalysis of reaction of alkynes with azides; see, for example, Crowley *et al.* (2010 ▸). Continuing our research concerning multifunctional chelating ligands in the construction of supra­molecular metal–organic frameworks, we used bis­(pyridyl­triazole) ligands to make macrocyclic Cu^II^ dimers that have found application in hosting small mol­ecules such as DABCO and oxalate (Pokharel *et al.*, 2013 ▸, 2014 ▸). As an extension of this work, we were also inter­ested in Ru^II^ pyridyl­triazole complexes. Ru^II^–polypyridine coordination compounds have been employed in dye-sensitized solar cells, optical sensors, and photoredox catalysts (Grätzel, 2009 ▸; Orellana & García-Fresnadillo, 2004 ▸; Prier *et al.*, 2013 ▸). In contrast, only a small number of Ru^II^–pyridyl­triazole complexes have been examined to ascertain whether incorporation of triazole could result in improvements compared to the polypyridine complexes. Triazole is a stronger π acceptor analog of pyridine, because of its three electronegative nitro­gen atoms, leading to Ru complexes with different photophysical and electrochemical properties (Schulze *et al.*, 2009 ▸; Felici *et al.*, 2009 ▸; Elliott *et al.*, 2016 ▸). Kumar *et al.* (2016 ▸) used benzyl­pyridytriazole (bpt, **1**) to synthesize the homoleptic Ru^II^ complex Ru(bpt)_3_^2+^.
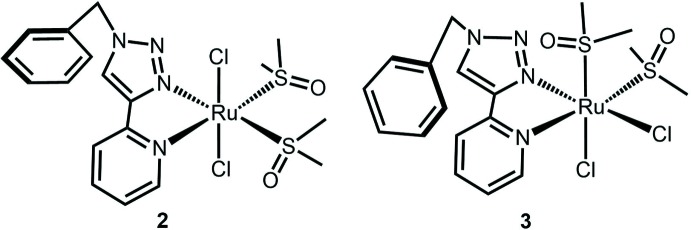


**Figure 1:**
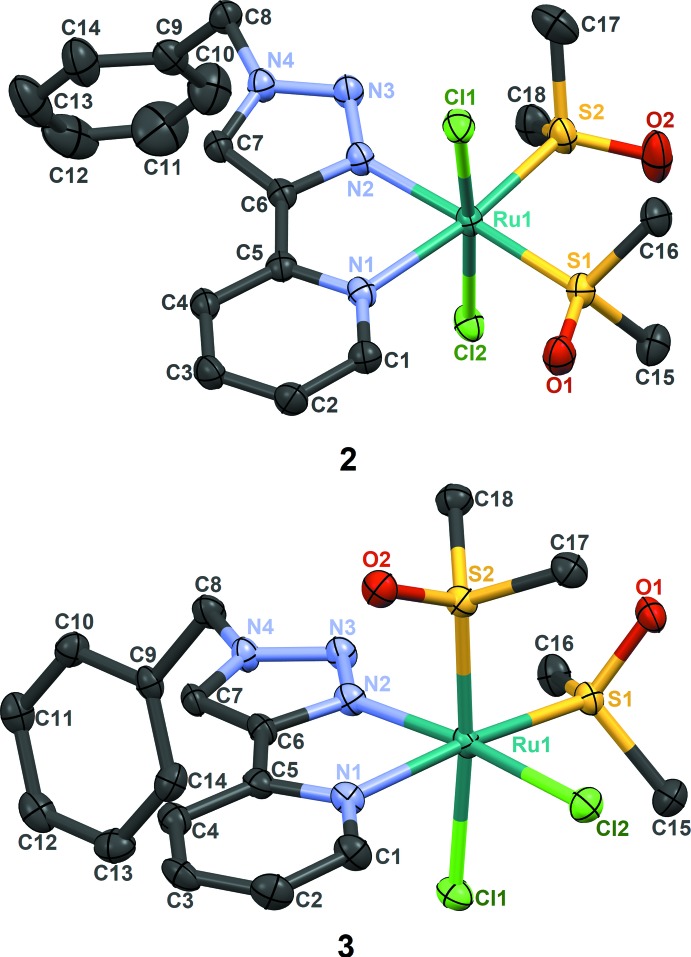
X-ray structures of **2** and **3.** Displacement ellipsoids are drawn at the 50% probability level, and hydrogen atoms are omitted for clarity.

Our intention was to make an Ru^II^ complex with one or two pyridyl­triazoles per metal atom along with weakly ligated coordination sites to facilitate other types of chemistry. In this paper, we report the synthesis of two isomers of Ru(bpt)(DMSO)_2_Cl_2_, **2** and **3** (see Fig. 1 ▸). Compound **2** is the kinetic product of the reaction, and it slowly isomerizes to the thermodynamically more stable **3**.

## Structural commentary   

Complexation of RuCl_2_(DMSO)_4_ and bpt in refluxing acetone gave compound **2** in good yield. Although enough bpt was present in the mixture to replace all DMSO mol­ecules, the product contains only one mol­ecule of bpt per Ru atom. The Ru^II^ cation in **2** adopts a distorted octa­hedral geometry and beside one bpt, two S-bonded DMSO mol­ecules occupy equatorial positions, and chlorides are coordinated in axial positions. This is the *OC*-6-14 isomer, according to Chemical Abstracts stereochemical notation (Brown *et al.*, 1975 ▸; Connelly & Damhus, 2005 ▸). The lengths of important bonds, the distances of the Ru atoms from the mean planes of the bpt ligands, and the angles between the pyridyl­triazole and benzyl mean planes, are reported in Table 1 ▸. We performed 2D NMR analysis to fully assign the peaks in the ^1^H and ^1^C NMR spectra. The HMBC spectrum shows cross coupling of H3, but not H2, with C5. This assignment, along with information from HSQC, NOESY, and COSY spectra (see supporting information), led to consistent assignments for the remaining atoms in **2**. In this structure, the DMSO mol­ecules are bonded through S, with S1—Ru1—S2 = 91.27 (2)°, and they are in slightly different environments, in agreement with the NMR data.

Compound **3**, the thermodynamically stable product of complexation of RuCl_2_(DMSO)_4_ and bpt, forms under reflux in toluene. During the reaction we detected **2** by ^1^H NMR as an inter­mediate, and it gradually isomerizes to **3**. The atoms *trans* to the two DMSO and chlorido ligands are similar or identical in **2**, but different in **3** (which is the *OC*-6-32 isomer). However, bond lengths and angles in **2** and **3** are only slightly different (see Table 1 ▸). The ^1^H NMR resonances for the two DMSO ligands differ by more in **3** (four singlet peaks) than they do in **2**, as expected. Unlike in **2**, the benzylic methyl­ene hydrogens in **3** are inequivalent, and they appear as a multiplet at 5.67 ppm.

Two other isomers of the title compound, with DMSO ligands *trans* and Cl ligands *cis* (the *OC*-6-43 isomer) or with *cis* DMSO and Cl ligands and pyridyl *trans* to Cl (*OC*-6-42), are possible. We did not observe any other materials in the NMR spectra or in the isolated products that were attributable to these isomers.

## Supra­molecular features   

The packing structure of **2** shows a non-classical hydrogen bond between Cl2 and H7 (see Table 2 ▸). The methine hydrogen (H7) is relatively acidic, showing a downfield ^1^H NMR peak at 7.93 ppm. Li & Flood (2008 ▸) took advantage of this C—H(triazole)⋯Cl inter­action in preparing a neutral, macrocyclic receptor for chloride ions. Hydrogen bonds to triazole H atoms were also used by White & Beer (2012 ▸) in creating a host system that can strongly bind halides. The packing structure of **3** also shows a close inter­action of H7, this time with O1 (see Table 3 ▸).

## Database survey   

A survey of the Cambridge Structural Database (version 5.40; Groom *et al.*, 2016 ▸) yielded 31 Ru complexes with pyridyl­triazole-based ligands. [Hits with bis­(triazol­yl)pyridine ligands were not included in the analysis.] All of the Ru centers in these structures have the +2 oxidation state and an approximately octa­hedral geometry. In these structures, the average N(pyridine)—Ru—N(triazole) angle, Ru—N(pyridine), and Ru—N(triazole) bond lengths are 78.4 (5)°, 2.088 (10) Å, and 2.040 (17) Å, respectively; the maximum deviation of Ru from the mean plane of the pyridyl­triazole ligand is 0.319 Å. The corresponding values for **2** and **3** are listed in Table 1 ▸, showing that their structural characteristics are similar to those of the reported structures in the literature.

## Synthesis and crystallization   

**General.** RuCl_3_·3H_2_O was purchased from Pressure Chemical; other reagents and solvents were purchased from Aldrich, Alfa Aesar, Acros Organics, or Combi-Blocks, and used without further purification. Bpt (**1**) was synthesized according to the procedure of Crowley *et al.* (2010 ▸) and purified by trituration with ether. The Ru starting material was *fac*-[RuCl_2_(DMSO-*S*)_3_(DMSO-*O*)], prepared following the literature procedure (Evans *et al.*, 1973 ▸) and characterized by comparison with the ^1^H NMR spectra of Bratsos & Alessio (2010 ▸). Elsewhere in this manuscript, it is referred to as RuCl_2_(DMSO)_4_ for simplicity. NMR spectra were recorded on a Bruker AV-400 MHz spectrometer and are reported in ppm, with coupling constants in Hz. Electrospray ionization mass spectra (ESI-MS) were measured on an Agilent 6210 instrument.

**Synthesis of (** ***OC*** **-6-14)-Ru(bpt)(DMSO)_2_Cl_2_, 2.** RuCl_2_(DMSO)_4_ (101.5 mg, 0.2095 mmol) and bpt (98.3 mg, 0.416 mmol) were mixed with 20 mL acetone and the mixture refluxed for 12 h under nitro­gen. The bright-yellow solution was allowed to cool to room temperature and the acetone evaporated *in vacuo*. Excess bpt was removed from the product as follows: The solid was sonicated with 5 mL of ether, the suspension centrifuged, and the solvent deca­nted. This process was repeated twice more. The resulting yellow solid was dried in air; yield 110 mg (93%). This material contains *ca* 95% **2** and 5% **3** by NMR. Yellow single crystals of **2** were obtained by vapor diffusion of ether into a solution of the complex in ethanol–chloro­form (1:1 *v*/*v*). ^1^H NMR (400 MHz, CDCl_3_) δ 10.59 (*d*, *J* = 5.04, H1), 7.93 (*s*, H7), 7.81 (*td*, *J*_1_ = 7.68 Hz, *J*_2_ = 1.32 Hz, H3), 7.64 (*d*, *J* = 7.56, H4), 7.46–7.51 (*m*, H2, H11, H12, H13), 7.35–7.39 (*m*, H10, H14), 5.65 (*s*, H8), 3.60 (*s*, DMSO), 3.58 (*s*, DMSO). ^13^C NMR (100 MHz, CDCl_3_) δ 155.64 (C1), 148.92, 148.82 (C5, C6), 137.37(C3), 131. 94 (C9), 129.90, 129.70 (C11/C13, C12), 128.84 (C10/C14), 124.73 (C2), 122.39 (C7), 120.77 (C4), 56.20 (C8), 46.42 (DMSO), 44.53 (DMSO). ESI–MS: *m*/*z* [Ru(bpt)(DMSO)_2_Cl_2_+Na]^+^ 580.9665 (calculated: 580.9686).

**Synthesis of (** ***OC*** **-6-32)-Ru(bpt)(DMSO)_2_Cl_2_, 3.** RuCl_2_(DMSO)_4_ (513.5 mg, 1.059 mmol) and bpt (361.5 mg, 1.530 mmol) were mixed with 15 mL toluene and the mixture refluxed for 16 days under nitro­gen, then cooled to room temperature. The resulting yellow suspension was filtered and the solid washed with fresh toluene and ether, then dried in air. Yield 590 mg (98%) of yellow solid **3**. For crystallization, a sample was mixed with aceto­nitrile, heated to boiling, allowed to cool, centrifuged, and the yellow deca­ntate used for ether vapor diffusion. After a day, yellow cube-shaped crystals were obtained. ^1^H NMR (400 MHz, CDCl_3_) δ 9.86 (*d*, *J* = 5.68, H1), 7.96 (*s*, H7), 7.87 (*td*, *J*_1_ = 7.68 Hz, *J*_2_ = 1.48 Hz, H3), 7.66 (*d*, *J =* 7.76 Hz, H4), 7.43–7.53 (*m*, H2, H12, H11, H13), 7.32–7.37 (*m*, H10, H14), 5.67 (*m*, H8), 3.69 (*s*, DMSO), 3.55 (*s*, DMSO), 3.12 (*s*, DMSO), 3.07 (*s*, DMSO). ^13^C NMR (100 MHz, DMSO-*d* ^6^) δ 152.02, 149.91, 149.52, 138.72, 135.27, 129.45, 129.20, 128.77, 125.69, 124.53, 121.39, 55.45, 46.55, 45.20, 44.70, 43.91. ESI–MS: *m*/*z* [Ru(bpt)(DMSO)_2_Cl_2_+Na]^+^ 580.9670 (calculated: 580.9686).

## Refinement   

Crystal data, data collection, and structure refinement details are summarized in Table 4 ▸. In both structures, H atoms were placed in idealized positions and treated with a riding model, with C—H distances of 0.95 Å for C*sp* ^2^, 0.99 Å for CH_2_, and 0.98 Å for methyl groups. *U*_iso_(H) values were set to either 1.2 or 1.5 (CH_3_) times *U*_eq_ of the attached atom. The largest peaks in the final difference maps of **2** and **3** are located 0.914 and 0.887 Å, respectively, from Ru1.

## Supplementary Material

Crystal structure: contains datablock(s) 2, 3. DOI: 10.1107/S2056989019008375/jj2212sup1.cif

Structure factors: contains datablock(s) 2. DOI: 10.1107/S2056989019008375/jj22122sup2.hkl

Supporting information file. DOI: 10.1107/S2056989019008375/jj22122sup4.cdx

Structure factors: contains datablock(s) 3. DOI: 10.1107/S2056989019008375/jj22123sup3.hkl

Supporting information file. DOI: 10.1107/S2056989019008375/jj22123sup5.cdx

1H NMR spectrum of complex 2. DOI: 10.1107/S2056989019008375/jj2212sup6.tif

13C NMR spectrum of complex 2. DOI: 10.1107/S2056989019008375/jj2212sup7.tif

HMBC spectrum of complex 2. DOI: 10.1107/S2056989019008375/jj2212sup8.tif

HSQC spectrum of complex 2. DOI: 10.1107/S2056989019008375/jj2212sup9.tif

NOESY spectrum of complex 2. DOI: 10.1107/S2056989019008375/jj2212sup10.tif

1H NMR spectrum of complex 2. DOI: 10.1107/S2056989019008375/jj2212sup11.tif

1H NMR spectrum of complex 3. DOI: 10.1107/S2056989019008375/jj2212sup12.tif

13C NMR spectrum of complex 3. DOI: 10.1107/S2056989019008375/jj2212sup13.tif

CCDC references: 1922625, 1922624

Additional supporting information:  crystallographic information; 3D view; checkCIF report

## Figures and Tables

**Table 1:** Selected bond distances for complexes **2** and **3**, the distance between Ru and the mean plane of the pyridyl­triazole (Å), the N1—Ru—N2 angle, and the angle between the pyridyl­triazole and benzyl mean planes (°)

	complex **2**	complex **3**
Ru1—N1 (pyridine)	2.1714 (18)	2.126 (3)
Ru1—N2 (triazole)	2.0890 (19)	2.044 (3)
Ru1—Cl1	2.3835 (6)	2.4175 (9)
Ru1—Cl2	2.4157 (6)	2.4167 (9)
Ru1—S1	2.2814 (6)	2.2530 (9)
Ru1—S2	2.2440 (6)	2.2434 (9)
Ru1⋯mean plane of pyridyl­triazole	0.0728 (2)	0.048 (3)
N1—Ru—N2	77.10 (7)	78.32 (12)
pyridyl­triazole plane⋯benzyl plane	77.75 (7)	69.52 (10)

**Table 2:** Hydrogen-bond geometry (Å, °) for **2** [Chem scheme1]

*D*—H⋯*A*	*D*—H	H⋯*A*	*D*⋯*A*	*D*—H⋯*A*
C7—H7⋯Cl2^i^	0.95	2.49	3.438 (2)	172

Symmetry code: (i) 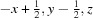.

**Table 3:** Hydrogen-bond geometry (Å, °) for **3** [Chem scheme1]

*D*—H⋯*A*	*D*—H	H⋯*A*	*D*⋯*A*	*D*—H⋯*A*
C7—H7⋯O1^i^	0.95	2.11	3.031 (4)	164

Symmetry code: (i) 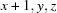.

**Table 4:** Experimental details

	**2**	**3**
Crystal data		
Chemical formula	[RuCl_2_(C_14_H_12_N_4_)(C_2_H_6_OS)_2_]	[RuCl_2_(C_14_H_12_N_4_)(C_2_H_6_OS)_2_]
*M*_r_	564.50	564.50
Crystal system, space group	Orthorhombic, *P* *b* *c* *a*	Triclinic, *P* 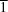
Temperature (K)	90	90
*a*, *b*, *c* (Å)	21.3094 (11), 9.4213 (5), 22.5267 (12)	9.3535 (14), 9.4900 (15), 13.904 (2)
α, β, γ (°)	90, 90, 90	98.893 (5), 106.772 (5), 106.276 (5)
*V* (Å^3^)	4522.5 (4)	1096.4 (3)
*Z*	8	2
Radiation type	Cu *K*α	Cu *K*α
μ (mm^−1^)	9.70	10.01
Crystal size (mm)	0.71 × 0.16 × 0.04	0.67 × 0.63 × 0.45

Data collection		
Diffractometer	Bruker Kappa APEXII CCD DUO	Bruker Kappa APEXII CCD DUO
Absorption correction	Multi-scan (*SADABS*; Krause *et al.*, 2015 ▸)	Multi-scan (*SADABS*; Krause *et al.*, 2015 ▸)
*T*_min_, *T*_max_	0.349, 0.715	0.062, 0.094
No. of measured, independent and observed [*I* > 2σ(*I*)] reflections	34572, 3970, 3628	9693, 3704, 3657
*R*_int_	0.043	0.026
(sin θ/λ)_max_ (Å^−1^)	0.596	0.596

Refinement		
*R*[*F* ^2^ > 2σ(*F* ^2^)], *wR*(*F* ^2^), *S*	0.025, 0.067, 1.04	0.037, 0.103, 1.16
No. of reflections	3970	3704
No. of parameters	266	266
H-atom treatment	H-atom parameters constrained	H-atom parameters constrained
Δρ_max_, Δρ_min_ (e Å^−3^)	0.60, −0.46	2.21, −0.63

Computer programs: *APEX3* (Bruker, 2016 ▸), *SAINT* (Bruker, 2012 ▸), *SHELXT2014* (Sheldrick, 2015*a* ▸), *SHELXL2017* (Sheldrick, 2015*b* ▸), *Mercury* (Macrae *et al.*, 2006 ▸) and *publCIF* (Westrip, 2010 ▸).

## supplementary crystallographic information

### (OC-6-14)-[1-Benzyl-4-(pyridin-2-yl-κN)-1H-1,2,3-triazole-κN3]dichloridobis(dimethyl sulfoxide-κS)ruthenium(II) (2) Crystal data

**Table:**

[RuCl_2_(C_14_H_12_N_4_)(C_2_H_6_OS)_2_]	*D*_x_ = 1.658 Mg m^−^^3^
*M**_r_* = 564.50	Cu *K*α radiation, λ = 1.54184 Å
Orthorhombic, *P**b**c**a*	Cell parameters from 9998 reflections
*a* = 21.3094 (11) Å	θ = 3.9–66.5°
*b* = 9.4213 (5) Å	µ = 9.70 mm^−^^1^
*c* = 22.5267 (12) Å	*T* = 90 K
*V* = 4522.5 (4) Å^3^	Needle, yellow
*Z* = 8	0.71 × 0.16 × 0.04 mm
*F*(000) = 2288	

### (OC-6-14)-[1-Benzyl-4-(pyridin-2-yl-κN)-1H-1,2,3-triazole-κN3]dichloridobis(dimethyl sulfoxide-κS)ruthenium(II) (2) Data collection

**Table:**

Bruker Kappa APEXII CCD DUO diffractometer	3970 independent reflections
Radiation source: IµS microfocus	3628 reflections with *I* > 2σ(*I*)
QUAZAR multilayer optics monochromator	*R*_int_ = 0.043
φ and ω scans	θ_max_ = 66.7°, θ_min_ = 3.9°
Absorption correction: multi-scan (SADABS; Krause et al., 2015)	*h* = −24→23
*T*_min_ = 0.349, *T*_max_ = 0.715	*k* = −11→9
34572 measured reflections	*l* = −26→26

### (OC-6-14)-[1-Benzyl-4-(pyridin-2-yl-κN)-1H-1,2,3-triazole-κN3]dichloridobis(dimethyl sulfoxide-κS)ruthenium(II) (2) Refinement

**Table:**

Refinement on *F*^2^	0 restraints
Least-squares matrix: full	Hydrogen site location: inferred from neighbouring sites
*R*[*F*^2^ > 2σ(*F*^2^)] = 0.025	H-atom parameters constrained
*wR*(*F*^2^) = 0.067	*w* = 1/[σ^2^(*F*_o_^2^) + (0.0391*P*)^2^ + 3.2772*P*] where *P* = (*F*_o_^2^ + 2*F*_c_^2^)/3
*S* = 1.04	(Δ/σ)_max_ = 0.002
3970 reflections	Δρ_max_ = 0.60 e Å^−^^3^
266 parameters	Δρ_min_ = −0.46 e Å^−^^3^

### (OC-6-14)-[1-Benzyl-4-(pyridin-2-yl-κN)-1H-1,2,3-triazole-κN3]dichloridobis(dimethyl sulfoxide-κS)ruthenium(II) (2) Special details

**Table:**

Geometry. All esds (except the esd in the dihedral angle between two l.s. planes) are estimated using the full covariance matrix. The cell esds are taken into account individually in the estimation of esds in distances, angles and torsion angles; correlations between esds in cell parameters are only used when they are defined by crystal symmetry. An approximate (isotropic) treatment of cell esds is used for estimating esds involving l.s. planes.

### (OC-6-14)-[1-Benzyl-4-(pyridin-2-yl-κN)-1H-1,2,3-triazole-κN3]dichloridobis(dimethyl sulfoxide-κS)ruthenium(II) (2) Fractional atomic coordinates and isotropic or equivalent isotropic displacement parameters (Å^2^)

**Table:**

	*x*	*y*	*z*	*U*_iso_*/*U*_eq_	
Ru1	0.09298 (2)	0.17864 (2)	0.37532 (2)	0.02143 (8)	
Cl1	0.08456 (3)	0.01442 (6)	0.45541 (2)	0.02897 (14)	
Cl2	0.11140 (3)	0.34903 (6)	0.29710 (2)	0.02744 (13)	
S1	0.04525 (3)	0.34440 (6)	0.43372 (2)	0.02548 (13)	
S2	0.00095 (3)	0.11879 (7)	0.33388 (3)	0.02991 (14)	
O2	−0.05488 (9)	0.2095 (2)	0.34285 (9)	0.0476 (5)	
O1	0.08383 (8)	0.4194 (2)	0.47853 (8)	0.0336 (4)	
N1	0.18911 (8)	0.2124 (2)	0.40402 (8)	0.0224 (4)	
N2	0.14267 (8)	0.0257 (2)	0.32734 (8)	0.0240 (4)	
N3	0.12683 (9)	−0.0753 (2)	0.28984 (8)	0.0264 (4)	
N4	0.18058 (9)	−0.1375 (2)	0.27335 (8)	0.0240 (4)	
C1	0.21032 (11)	0.3039 (2)	0.44464 (10)	0.0261 (5)	
H1	0.180986	0.362842	0.464654	0.031*	
C2	0.27331 (12)	0.3162 (3)	0.45875 (11)	0.0301 (5)	
H2	0.286609	0.383822	0.487405	0.036*	
C3	0.31690 (11)	0.2297 (3)	0.43097 (11)	0.0297 (5)	
H3	0.360309	0.237149	0.440043	0.036*	
C4	0.29572 (11)	0.1328 (3)	0.38988 (10)	0.0256 (5)	
H4	0.324292	0.070938	0.370413	0.031*	
C5	0.23209 (11)	0.1268 (3)	0.37732 (9)	0.0230 (5)	
C6	0.20593 (10)	0.0266 (2)	0.33525 (9)	0.0223 (5)	
C7	0.23052 (10)	−0.0784 (2)	0.30006 (9)	0.0235 (4)	
H7	0.273411	−0.103661	0.295568	0.028*	
C8	0.18019 (11)	−0.2548 (3)	0.23009 (10)	0.0281 (5)	
H8A	0.136727	−0.271837	0.216302	0.034*	
H8AB	0.195534	−0.342585	0.249364	0.034*	
C18	0.00697 (12)	0.1011 (3)	0.25524 (11)	0.0365 (6)	
H18A	−0.033416	0.069712	0.239103	0.055*	
H18B	0.039385	0.031020	0.245612	0.055*	
H18C	0.018252	0.192946	0.237803	0.055*	
C17	−0.02254 (14)	−0.0564 (3)	0.35314 (13)	0.0459 (7)	
H17A	−0.058689	−0.084239	0.328895	0.069*	
H17B	−0.034137	−0.059392	0.395224	0.069*	
H17C	0.012264	−0.122196	0.345925	0.069*	
C9	0.22158 (12)	−0.2194 (3)	0.17743 (10)	0.0317 (5)	
C10	0.19814 (17)	−0.1376 (4)	0.13231 (13)	0.0518 (8)	
H10	0.155735	−0.106577	0.133446	0.062*	
C11	0.2363 (2)	−0.1000 (4)	0.08503 (15)	0.0686 (11)	
H11	0.219937	−0.043315	0.053763	0.082*	
C12	0.29860 (18)	−0.1453 (4)	0.08322 (15)	0.0614 (10)	
H12	0.325403	−0.115599	0.051885	0.074*	
C13	0.32091 (16)	−0.2331 (5)	0.12711 (14)	0.0592 (10)	
H13	0.362443	−0.269291	0.124741	0.071*	
C14	0.28276 (13)	−0.2694 (4)	0.17521 (13)	0.0476 (7)	
H14	0.298593	−0.327775	0.206148	0.057*	
C15	0.00646 (13)	0.4811 (3)	0.39304 (11)	0.0349 (6)	
H15A	0.037764	0.541872	0.373815	0.052*	
H15B	−0.019102	0.538224	0.420192	0.052*	
H15C	−0.020607	0.438318	0.362755	0.052*	
C16	−0.02029 (12)	0.2740 (3)	0.47367 (11)	0.0336 (5)	
H16A	−0.050315	0.232593	0.445651	0.050*	
H16B	−0.040715	0.350377	0.496027	0.050*	
H16C	−0.005731	0.200585	0.501229	0.050*	

### (OC-6-14)-[1-Benzyl-4-(pyridin-2-yl-κN)-1H-1,2,3-triazole-κN3]dichloridobis(dimethyl sulfoxide-κS)ruthenium(II) (2) Atomic displacement parameters (Å^2^)

**Table:**

	*U*^11^	*U*^22^	*U*^33^	*U*^12^	*U*^13^	*U*^23^
Ru1	0.01644 (12)	0.02583 (12)	0.02202 (11)	0.00089 (6)	0.00003 (6)	−0.00100 (6)
Cl1	0.0250 (3)	0.0305 (3)	0.0313 (3)	0.0012 (2)	0.0041 (2)	0.0048 (2)
Cl2	0.0217 (3)	0.0355 (3)	0.0251 (3)	0.0012 (2)	−0.0010 (2)	0.0042 (2)
S1	0.0226 (3)	0.0286 (3)	0.0251 (3)	0.0031 (2)	0.0023 (2)	−0.0011 (2)
S2	0.0197 (3)	0.0379 (3)	0.0321 (3)	0.0011 (2)	−0.0021 (2)	−0.0057 (2)
O2	0.0265 (9)	0.0660 (13)	0.0503 (12)	0.0126 (9)	−0.0099 (8)	−0.0207 (10)
O1	0.0288 (9)	0.0383 (10)	0.0337 (9)	0.0039 (7)	0.0007 (7)	−0.0093 (8)
N1	0.0207 (9)	0.0259 (10)	0.0207 (9)	−0.0012 (7)	0.0015 (7)	0.0031 (7)
N2	0.0191 (9)	0.0286 (10)	0.0245 (9)	0.0002 (7)	−0.0009 (7)	−0.0011 (8)
N3	0.0218 (10)	0.0306 (11)	0.0268 (9)	0.0013 (8)	−0.0001 (7)	−0.0046 (8)
N4	0.0216 (9)	0.0277 (10)	0.0226 (9)	0.0011 (8)	0.0015 (7)	−0.0017 (8)
C1	0.0265 (12)	0.0273 (12)	0.0247 (11)	−0.0001 (9)	−0.0007 (9)	−0.0005 (9)
C2	0.0267 (13)	0.0300 (13)	0.0336 (13)	−0.0012 (9)	−0.0051 (10)	−0.0039 (10)
C3	0.0212 (11)	0.0316 (13)	0.0364 (12)	−0.0021 (10)	−0.0051 (9)	0.0017 (10)
C4	0.0198 (11)	0.0278 (12)	0.0293 (11)	0.0014 (9)	0.0001 (9)	0.0037 (10)
C5	0.0237 (12)	0.0230 (11)	0.0223 (11)	0.0006 (9)	0.0009 (8)	0.0043 (8)
C6	0.0191 (11)	0.0262 (12)	0.0217 (10)	−0.0002 (9)	−0.0001 (8)	0.0035 (9)
C7	0.0191 (11)	0.0270 (12)	0.0244 (10)	0.0002 (9)	0.0006 (8)	0.0019 (9)
C8	0.0272 (12)	0.0303 (13)	0.0269 (11)	0.0000 (10)	0.0001 (9)	−0.0064 (10)
C18	0.0297 (13)	0.0474 (15)	0.0325 (13)	−0.0005 (11)	−0.0077 (10)	−0.0054 (11)
C17	0.0371 (15)	0.0541 (18)	0.0465 (16)	−0.0165 (13)	−0.0054 (13)	0.0020 (14)
C9	0.0311 (13)	0.0375 (13)	0.0265 (12)	−0.0047 (11)	0.0033 (10)	−0.0103 (10)
C10	0.055 (2)	0.063 (2)	0.0378 (15)	0.0074 (16)	0.0089 (13)	0.0044 (14)
C11	0.090 (3)	0.072 (3)	0.0439 (18)	0.000 (2)	0.0190 (18)	0.0109 (17)
C12	0.071 (2)	0.069 (2)	0.0434 (18)	−0.0280 (19)	0.0284 (17)	−0.0162 (16)
C13	0.0360 (17)	0.086 (3)	0.055 (2)	−0.0121 (17)	0.0139 (13)	−0.0219 (19)
C14	0.0331 (15)	0.065 (2)	0.0445 (15)	0.0003 (14)	0.0061 (12)	−0.0093 (15)
C15	0.0346 (14)	0.0381 (15)	0.0319 (12)	0.0091 (11)	0.0035 (11)	0.0016 (11)
C16	0.0276 (12)	0.0384 (14)	0.0348 (12)	0.0010 (11)	0.0108 (10)	−0.0016 (11)

### (OC-6-14)-[1-Benzyl-4-(pyridin-2-yl-κN)-1H-1,2,3-triazole-κN3]dichloridobis(dimethyl sulfoxide-κS)ruthenium(II) (2) Geometric parameters (Å, º)

**Table:**

Ru1—N2	2.0890 (19)	C6—C7	1.371 (3)
Ru1—N1	2.1714 (18)	C7—H7	0.9500
Ru1—S2	2.2440 (6)	C8—C9	1.515 (3)
Ru1—S1	2.2814 (6)	C8—H8A	0.9900
Ru1—Cl1	2.3835 (6)	C8—H8AB	0.9900
Ru1—Cl2	2.4157 (6)	C18—H18A	0.9800
S1—O1	1.4814 (18)	C18—H18B	0.9800
S1—C15	1.784 (3)	C18—H18C	0.9800
S1—C16	1.789 (2)	C17—H17A	0.9800
S2—O2	1.4786 (19)	C17—H17B	0.9800
S2—C17	1.779 (3)	C17—H17C	0.9800
S2—C18	1.784 (3)	C9—C10	1.370 (4)
N1—C1	1.336 (3)	C9—C14	1.387 (4)
N1—C5	1.360 (3)	C10—C11	1.386 (5)
N2—N3	1.317 (3)	C10—H10	0.9500
N2—C6	1.360 (3)	C11—C12	1.396 (6)
N3—N4	1.339 (3)	C11—H11	0.9500
N4—C7	1.344 (3)	C12—C13	1.374 (6)
N4—C8	1.473 (3)	C12—H12	0.9500
C1—C2	1.384 (4)	C13—C14	1.397 (4)
C1—H1	0.9500	C13—H13	0.9500
C2—C3	1.385 (4)	C14—H14	0.9500
C2—H2	0.9500	C15—H15A	0.9800
C3—C4	1.376 (4)	C15—H15B	0.9800
C3—H3	0.9500	C15—H15C	0.9800
C4—C5	1.386 (3)	C16—H16A	0.9800
C4—H4	0.9500	C16—H16B	0.9800
C5—C6	1.449 (3)	C16—H16C	0.9800
			
N2—Ru1—N1	77.10 (7)	N2—C6—C5	118.1 (2)
N2—Ru1—S2	93.13 (5)	C7—C6—C5	134.5 (2)
N1—Ru1—S2	170.10 (5)	N4—C7—C6	104.79 (19)
N2—Ru1—S1	175.11 (5)	N4—C7—H7	127.6
N1—Ru1—S1	98.55 (5)	C6—C7—H7	127.6
S2—Ru1—S1	91.27 (2)	N4—C8—C9	110.5 (2)
N2—Ru1—Cl1	88.98 (5)	N4—C8—H8A	109.6
N1—Ru1—Cl1	86.60 (5)	C9—C8—H8A	109.6
S2—Ru1—Cl1	94.93 (2)	N4—C8—H8AB	109.6
S1—Ru1—Cl1	88.52 (2)	C9—C8—H8AB	109.6
N2—Ru1—Cl2	89.92 (5)	H8A—C8—H8AB	108.1
N1—Ru1—Cl2	88.09 (5)	S2—C18—H18A	109.5
S2—Ru1—Cl2	90.32 (2)	S2—C18—H18B	109.5
S1—Ru1—Cl2	92.19 (2)	H18A—C18—H18B	109.5
Cl1—Ru1—Cl2	174.69 (2)	S2—C18—H18C	109.5
O1—S1—C15	105.23 (12)	H18A—C18—H18C	109.5
O1—S1—C16	105.50 (11)	H18B—C18—H18C	109.5
C15—S1—C16	99.46 (13)	S2—C17—H17A	109.5
O1—S1—Ru1	118.17 (7)	S2—C17—H17B	109.5
C15—S1—Ru1	113.87 (9)	H17A—C17—H17B	109.5
C16—S1—Ru1	112.62 (9)	S2—C17—H17C	109.5
O2—S2—C17	106.05 (14)	H17A—C17—H17C	109.5
O2—S2—C18	104.33 (12)	H17B—C17—H17C	109.5
C17—S2—C18	100.13 (14)	C10—C9—C14	120.5 (3)
O2—S2—Ru1	120.08 (8)	C10—C9—C8	119.5 (2)
C17—S2—Ru1	112.19 (10)	C14—C9—C8	120.1 (3)
C18—S2—Ru1	111.94 (9)	C9—C10—C11	120.0 (3)
C1—N1—C5	117.23 (19)	C9—C10—H10	120.0
C1—N1—Ru1	128.13 (16)	C11—C10—H10	120.0
C5—N1—Ru1	114.63 (15)	C10—C11—C12	120.2 (4)
N3—N2—C6	110.02 (18)	C10—C11—H11	119.9
N3—N2—Ru1	134.47 (14)	C12—C11—H11	119.9
C6—N2—Ru1	115.51 (15)	C13—C12—C11	119.5 (3)
N2—N3—N4	105.96 (17)	C13—C12—H12	120.3
N3—N4—C7	111.83 (18)	C11—C12—H12	120.3
N3—N4—C8	120.44 (18)	C12—C13—C14	120.3 (3)
C7—N4—C8	127.72 (19)	C12—C13—H13	119.9
N1—C1—C2	122.6 (2)	C14—C13—H13	119.9
N1—C1—H1	118.7	C9—C14—C13	119.5 (3)
C2—C1—H1	118.7	C9—C14—H14	120.3
C1—C2—C3	119.8 (2)	C13—C14—H14	120.3
C1—C2—H2	120.1	S1—C15—H15A	109.5
C3—C2—H2	120.1	S1—C15—H15B	109.5
C4—C3—C2	118.3 (2)	H15A—C15—H15B	109.5
C4—C3—H3	120.8	S1—C15—H15C	109.5
C2—C3—H3	120.8	H15A—C15—H15C	109.5
C3—C4—C5	119.0 (2)	H15B—C15—H15C	109.5
C3—C4—H4	120.5	S1—C16—H16A	109.5
C5—C4—H4	120.5	S1—C16—H16B	109.5
N1—C5—C4	123.0 (2)	H16A—C16—H16B	109.5
N1—C5—C6	114.6 (2)	S1—C16—H16C	109.5
C4—C5—C6	122.4 (2)	H16A—C16—H16C	109.5
N2—C6—C7	107.39 (19)	H16B—C16—H16C	109.5
			
C6—N2—N3—N4	−0.8 (2)	C4—C5—C6—N2	177.9 (2)
Ru1—N2—N3—N4	179.26 (15)	N1—C5—C6—C7	−178.5 (2)
N2—N3—N4—C7	0.4 (2)	C4—C5—C6—C7	0.5 (4)
N2—N3—N4—C8	−178.75 (19)	N3—N4—C7—C6	0.1 (2)
C5—N1—C1—C2	1.7 (3)	C8—N4—C7—C6	179.2 (2)
Ru1—N1—C1—C2	−179.42 (18)	N2—C6—C7—N4	−0.6 (2)
N1—C1—C2—C3	−1.1 (4)	C5—C6—C7—N4	177.1 (2)
C1—C2—C3—C4	−0.2 (4)	N3—N4—C8—C9	123.2 (2)
C2—C3—C4—C5	0.9 (4)	C7—N4—C8—C9	−55.9 (3)
C1—N1—C5—C4	−1.0 (3)	N4—C8—C9—C10	−82.9 (3)
Ru1—N1—C5—C4	179.97 (17)	N4—C8—C9—C14	96.9 (3)
C1—N1—C5—C6	177.95 (19)	C14—C9—C10—C11	−2.3 (5)
Ru1—N1—C5—C6	−1.1 (2)	C8—C9—C10—C11	177.5 (3)
C3—C4—C5—N1	−0.3 (3)	C9—C10—C11—C12	0.0 (6)
C3—C4—C5—C6	−179.2 (2)	C10—C11—C12—C13	3.2 (6)
N3—N2—C6—C7	0.9 (2)	C11—C12—C13—C14	−4.1 (5)
Ru1—N2—C6—C7	−179.16 (14)	C10—C9—C14—C13	1.4 (5)
N3—N2—C6—C5	−177.21 (18)	C8—C9—C14—C13	−178.4 (3)
Ru1—N2—C6—C5	2.7 (2)	C12—C13—C14—C9	1.9 (5)
N1—C5—C6—N2	−1.1 (3)		

### (OC-6-14)-[1-Benzyl-4-(pyridin-2-yl-κN)-1H-1,2,3-triazole-κN3]dichloridobis(dimethyl sulfoxide-κS)ruthenium(II) (2) Hydrogen-bond geometry (Å, º)

**Table:**

*D*—H···*A*	*D*—H	H···*A*	*D*···*A*	*D*—H···*A*
C7—H7···Cl2^i^	0.95	2.49	3.438 (2)	172

Symmetry code: (i) −*x*+1/2, *y*−1/2, *z*.

### (OC-6-32)-[1-Benzyl-4-(pyridin-2-yl-κN)-1H-1,2,3-triazole-κN3]dichloridobis(dimethyl sulfoxide-κS)ruthenium(II) (3) Crystal data

**Table:**

[RuCl_2_(C_14_H_12_N_4_)(C_2_H_6_OS)_2_]	*Z* = 2
*M**_r_* = 564.50	*F*(000) = 572
Triclinic, *P*1	*D*_x_ = 1.710 Mg m^−^^3^
*a* = 9.3535 (14) Å	Cu *K*α radiation, λ = 1.54184 Å
*b* = 9.4900 (15) Å	Cell parameters from 7943 reflections
*c* = 13.904 (2) Å	θ = 3.4–66.9°
α = 98.893 (5)°	µ = 10.01 mm^−^^1^
β = 106.772 (5)°	*T* = 90 K
γ = 106.276 (5)°	Cubic, yellow
*V* = 1096.4 (3) Å^3^	0.67 × 0.63 × 0.45 mm

### (OC-6-32)-[1-Benzyl-4-(pyridin-2-yl-κN)-1H-1,2,3-triazole-κN3]dichloridobis(dimethyl sulfoxide-κS)ruthenium(II) (3) Data collection

**Table:**

Bruker Kappa APEXII CCD DUO diffractometer	3704 independent reflections
Radiation source: IµS microfocus	3657 reflections with *I* > 2σ(*I*)
QUAZAR multilayer optics monochromator	*R*_int_ = 0.026
φ and ω scans	θ_max_ = 66.9°, θ_min_ = 3.4°
Absorption correction: multi-scan (SADABS; Krause et al., 2015)	*h* = −10→11
*T*_min_ = 0.062, *T*_max_ = 0.094	*k* = −10→11
9693 measured reflections	*l* = −16→11

### (OC-6-32)-[1-Benzyl-4-(pyridin-2-yl-κN)-1H-1,2,3-triazole-κN3]dichloridobis(dimethyl sulfoxide-κS)ruthenium(II) (3) Refinement

**Table:**

Refinement on *F*^2^	0 restraints
Least-squares matrix: full	Hydrogen site location: inferred from neighbouring sites
*R*[*F*^2^ > 2σ(*F*^2^)] = 0.037	H-atom parameters constrained
*wR*(*F*^2^) = 0.103	*w* = 1/[σ^2^(*F*_o_^2^) + (0.0546*P*)^2^ + 2.1529*P*] where *P* = (*F*_o_^2^ + 2*F*_c_^2^)/3
*S* = 1.16	(Δ/σ)_max_ = 0.001
3704 reflections	Δρ_max_ = 2.21 e Å^−^^3^
266 parameters	Δρ_min_ = −0.63 e Å^−^^3^

### (OC-6-32)-[1-Benzyl-4-(pyridin-2-yl-κN)-1H-1,2,3-triazole-κN3]dichloridobis(dimethyl sulfoxide-κS)ruthenium(II) (3) Special details

**Table:**

Geometry. All esds (except the esd in the dihedral angle between two l.s. planes) are estimated using the full covariance matrix. The cell esds are taken into account individually in the estimation of esds in distances, angles and torsion angles; correlations between esds in cell parameters are only used when they are defined by crystal symmetry. An approximate (isotropic) treatment of cell esds is used for estimating esds involving l.s. planes.

### (OC-6-32)-[1-Benzyl-4-(pyridin-2-yl-κN)-1H-1,2,3-triazole-κN3]dichloridobis(dimethyl sulfoxide-κS)ruthenium(II) (3) Fractional atomic coordinates and isotropic or equivalent isotropic displacement parameters (Å^2^)

**Table:**

	*x*	*y*	*z*	*U*_iso_*/*U*_eq_	
Ru1	0.34993 (3)	0.83627 (3)	0.70658 (2)	0.01710 (13)	
Cl1	0.47441 (11)	0.82110 (10)	0.88125 (7)	0.0249 (2)	
C1	0.4322 (5)	0.5485 (4)	0.6392 (3)	0.0251 (8)	
H1	0.326454	0.492417	0.631964	0.030*	
C3	0.6834 (5)	0.5504 (4)	0.6309 (3)	0.0249 (8)	
H3	0.751793	0.498296	0.617920	0.030*	
N3	0.6204 (4)	1.1465 (4)	0.7637 (3)	0.0211 (6)	
C5	0.6319 (4)	0.7785 (4)	0.6771 (3)	0.0201 (7)	
S1	0.22838 (10)	0.98237 (10)	0.76998 (7)	0.0195 (2)	
S2	0.25606 (10)	0.85272 (10)	0.54247 (7)	0.0201 (2)	
Cl2	0.11642 (10)	0.61922 (10)	0.67424 (7)	0.0240 (2)	
C2	0.5301 (5)	0.4707 (4)	0.6218 (3)	0.0281 (8)	
H2	0.492296	0.362796	0.603645	0.034*	
N2	0.5625 (4)	0.9976 (3)	0.7295 (2)	0.0197 (6)	
O1	0.0918 (3)	1.0056 (3)	0.6943 (2)	0.0237 (6)	
O2	0.3217 (3)	0.7874 (3)	0.4691 (2)	0.0248 (6)	
N1	0.4805 (4)	0.7007 (3)	0.6660 (2)	0.0197 (6)	
N4	0.7735 (4)	1.1863 (3)	0.7691 (2)	0.0198 (6)	
C4	0.7360 (5)	0.7070 (4)	0.6590 (3)	0.0227 (8)	
H4	0.841005	0.764702	0.665853	0.027*	
C6	0.6756 (4)	0.9424 (4)	0.7130 (3)	0.0186 (7)	
C7	0.8119 (4)	1.0644 (4)	0.7384 (3)	0.0193 (7)	
H7	0.911618	1.063422	0.735085	0.023*	
C8	0.8771 (4)	1.3460 (4)	0.8080 (3)	0.0227 (8)	
H8A	0.818548	1.408213	0.831416	0.027*	
H8B	0.907011	1.381665	0.750905	0.027*	
C9	1.0257 (4)	1.3673 (4)	0.8971 (3)	0.0197 (7)	
C10	1.1666 (5)	1.4801 (4)	0.9085 (3)	0.0231 (8)	
H10	1.168318	1.539792	0.859658	0.028*	
C13	1.1646 (5)	1.3045 (5)	1.0502 (3)	0.0282 (9)	
H13	1.163861	1.242858	1.097911	0.034*	
C11	1.3043 (5)	1.5055 (4)	0.9911 (3)	0.0254 (8)	
H11	1.399325	1.583829	0.999096	0.030*	
C12	1.3051 (5)	1.4179 (5)	1.0621 (3)	0.0272 (8)	
H12	1.400003	1.435062	1.118164	0.033*	
C15	0.1582 (5)	0.9144 (5)	0.8671 (3)	0.0285 (8)	
H15A	0.104786	0.979669	0.892022	0.043*	
H15B	0.248383	0.916245	0.925322	0.043*	
H15C	0.083077	0.810137	0.837040	0.043*	
C14	1.0261 (5)	1.2809 (4)	0.9692 (3)	0.0244 (8)	
H14	0.930278	1.204961	0.962791	0.029*	
C16	0.3581 (5)	1.1661 (4)	0.8480 (3)	0.0254 (8)	
H16A	0.403859	1.224247	0.805103	0.038*	
H16B	0.443670	1.156519	0.904175	0.038*	
H16C	0.298350	1.218801	0.877788	0.038*	
C17	0.0452 (5)	0.7802 (4)	0.4819 (3)	0.0255 (8)	
H17A	0.015495	0.804549	0.414508	0.038*	
H17B	−0.002664	0.826346	0.526319	0.038*	
H17C	0.006768	0.669736	0.471409	0.038*	
C18	0.2932 (5)	1.0468 (4)	0.5377 (3)	0.0250 (8)	
H18A	0.238266	1.050697	0.467030	0.037*	
H18B	0.407723	1.098936	0.557126	0.037*	
H18C	0.253771	1.096943	0.586229	0.037*	

### (OC-6-32)-[1-Benzyl-4-(pyridin-2-yl-κN)-1H-1,2,3-triazole-κN3]dichloridobis(dimethyl sulfoxide-κS)ruthenium(II) (3) Atomic displacement parameters (Å^2^)

**Table:**

	*U*^11^	*U*^22^	*U*^33^	*U*^12^	*U*^13^	*U*^23^
Ru1	0.01527 (18)	0.01563 (18)	0.01954 (18)	0.00614 (12)	0.00501 (12)	0.00265 (12)
Cl1	0.0253 (5)	0.0278 (5)	0.0212 (4)	0.0119 (4)	0.0045 (4)	0.0065 (4)
C1	0.026 (2)	0.0198 (19)	0.0272 (19)	0.0087 (16)	0.0064 (16)	0.0024 (15)
C3	0.030 (2)	0.0251 (19)	0.0220 (18)	0.0183 (17)	0.0048 (16)	0.0033 (15)
N3	0.0177 (15)	0.0198 (16)	0.0245 (15)	0.0066 (12)	0.0062 (12)	0.0042 (12)
C5	0.0216 (18)	0.0227 (19)	0.0171 (17)	0.0106 (15)	0.0055 (14)	0.0055 (14)
S1	0.0180 (4)	0.0210 (4)	0.0202 (4)	0.0082 (3)	0.0070 (3)	0.0037 (3)
S2	0.0211 (4)	0.0185 (4)	0.0203 (4)	0.0076 (3)	0.0063 (3)	0.0036 (3)
Cl2	0.0215 (4)	0.0200 (4)	0.0269 (4)	0.0035 (3)	0.0074 (4)	0.0050 (3)
C2	0.033 (2)	0.0187 (18)	0.029 (2)	0.0088 (16)	0.0065 (17)	0.0027 (15)
N2	0.0198 (15)	0.0174 (15)	0.0207 (15)	0.0066 (12)	0.0061 (12)	0.0028 (12)
O1	0.0199 (13)	0.0305 (14)	0.0233 (13)	0.0134 (11)	0.0072 (11)	0.0055 (11)
O2	0.0282 (14)	0.0210 (13)	0.0255 (13)	0.0094 (11)	0.0106 (11)	0.0030 (11)
N1	0.0196 (15)	0.0180 (15)	0.0192 (15)	0.0071 (12)	0.0041 (12)	0.0025 (12)
N4	0.0139 (14)	0.0192 (15)	0.0237 (15)	0.0044 (12)	0.0052 (12)	0.0034 (12)
C4	0.0232 (19)	0.027 (2)	0.0197 (17)	0.0120 (16)	0.0070 (15)	0.0048 (15)
C6	0.0184 (17)	0.0201 (18)	0.0183 (16)	0.0099 (14)	0.0057 (14)	0.0029 (14)
C7	0.0150 (17)	0.0234 (18)	0.0221 (17)	0.0101 (14)	0.0070 (14)	0.0055 (14)
C8	0.0204 (18)	0.0178 (18)	0.0292 (19)	0.0069 (15)	0.0072 (16)	0.0062 (15)
C9	0.0175 (17)	0.0180 (17)	0.0232 (18)	0.0084 (14)	0.0064 (15)	0.0014 (14)
C10	0.0242 (19)	0.0185 (18)	0.0268 (19)	0.0060 (15)	0.0106 (16)	0.0052 (15)
C13	0.035 (2)	0.028 (2)	0.0224 (19)	0.0129 (18)	0.0102 (17)	0.0073 (16)
C11	0.0207 (19)	0.0211 (19)	0.029 (2)	0.0053 (15)	0.0064 (16)	−0.0007 (15)
C12	0.024 (2)	0.028 (2)	0.0235 (19)	0.0101 (16)	0.0021 (16)	−0.0026 (16)
C15	0.029 (2)	0.033 (2)	0.029 (2)	0.0115 (18)	0.0154 (17)	0.0117 (17)
C14	0.0238 (19)	0.0244 (19)	0.0242 (19)	0.0054 (16)	0.0114 (16)	0.0037 (15)
C16	0.025 (2)	0.0235 (19)	0.0262 (19)	0.0094 (16)	0.0094 (16)	−0.0014 (15)
C17	0.0237 (19)	0.025 (2)	0.0231 (18)	0.0073 (16)	0.0039 (15)	0.0036 (15)
C18	0.029 (2)	0.0223 (19)	0.0256 (19)	0.0100 (16)	0.0104 (16)	0.0071 (15)

### (OC-6-32)-[1-Benzyl-4-(pyridin-2-yl-κN)-1H-1,2,3-triazole-κN3]dichloridobis(dimethyl sulfoxide-κS)ruthenium(II) (3) Geometric parameters (Å, º)

**Table:**

Ru1—N2	2.044 (3)	C6—C7	1.370 (5)
Ru1—N1	2.126 (3)	C7—H7	0.9500
Ru1—S2	2.2434 (9)	C8—C9	1.509 (5)
Ru1—S1	2.2530 (9)	C8—H8A	0.9900
Ru1—Cl2	2.4167 (9)	C8—H8B	0.9900
Ru1—Cl1	2.4175 (9)	C9—C14	1.390 (5)
C1—N1	1.341 (5)	C9—C10	1.394 (5)
C1—C2	1.377 (6)	C10—C11	1.387 (6)
C1—H1	0.9500	C10—H10	0.9500
C3—C4	1.379 (6)	C13—C14	1.384 (6)
C3—C2	1.380 (6)	C13—C12	1.393 (6)
C3—H3	0.9500	C13—H13	0.9500
N3—N2	1.315 (4)	C11—C12	1.385 (6)
N3—N4	1.351 (4)	C11—H11	0.9500
C5—N1	1.351 (5)	C12—H12	0.9500
C5—C4	1.390 (5)	C15—H15A	0.9800
C5—C6	1.456 (5)	C15—H15B	0.9800
S1—O1	1.497 (3)	C15—H15C	0.9800
S1—C16	1.777 (4)	C14—H14	0.9500
S1—C15	1.793 (4)	C16—H16A	0.9800
S2—O2	1.477 (3)	C16—H16B	0.9800
S2—C17	1.782 (4)	C16—H16C	0.9800
S2—C18	1.792 (4)	C17—H17A	0.9800
C2—H2	0.9500	C17—H17B	0.9800
N2—C6	1.364 (5)	C17—H17C	0.9800
N4—C7	1.348 (5)	C18—H18A	0.9800
N4—C8	1.466 (5)	C18—H18B	0.9800
C4—H4	0.9500	C18—H18C	0.9800
			
N2—Ru1—N1	78.32 (12)	N2—C6—C5	117.9 (3)
N2—Ru1—S2	90.28 (9)	C7—C6—C5	135.0 (3)
N1—Ru1—S2	91.60 (8)	N4—C7—C6	105.0 (3)
N2—Ru1—S1	100.24 (9)	N4—C7—H7	127.5
N1—Ru1—S1	173.01 (8)	C6—C7—H7	127.5
S2—Ru1—S1	95.26 (3)	N4—C8—C9	111.4 (3)
N2—Ru1—Cl2	171.78 (9)	N4—C8—H8A	109.3
N1—Ru1—Cl2	93.49 (9)	C9—C8—H8A	109.3
S2—Ru1—Cl2	90.60 (3)	N4—C8—H8B	109.3
S1—Ru1—Cl2	87.81 (3)	C9—C8—H8B	109.3
N2—Ru1—Cl1	85.57 (9)	H8A—C8—H8B	108.0
N1—Ru1—Cl1	84.32 (8)	C14—C9—C10	119.0 (3)
S2—Ru1—Cl1	174.69 (3)	C14—C9—C8	122.3 (3)
S1—Ru1—Cl1	88.75 (3)	C10—C9—C8	118.7 (3)
Cl2—Ru1—Cl1	93.04 (3)	C11—C10—C9	120.1 (4)
N1—C1—C2	122.5 (4)	C11—C10—H10	119.9
N1—C1—H1	118.8	C9—C10—H10	119.9
C2—C1—H1	118.8	C14—C13—C12	120.4 (4)
C4—C3—C2	119.0 (4)	C14—C13—H13	119.8
C4—C3—H3	120.5	C12—C13—H13	119.8
C2—C3—H3	120.5	C12—C11—C10	120.8 (4)
N2—N3—N4	105.3 (3)	C12—C11—H11	119.6
N1—C5—C4	122.5 (3)	C10—C11—H11	119.6
N1—C5—C6	113.6 (3)	C11—C12—C13	119.0 (4)
C4—C5—C6	123.8 (3)	C11—C12—H12	120.5
O1—S1—C16	106.23 (18)	C13—C12—H12	120.5
O1—S1—C15	106.64 (18)	S1—C15—H15A	109.5
C16—S1—C15	97.9 (2)	S1—C15—H15B	109.5
O1—S1—Ru1	117.75 (11)	H15A—C15—H15B	109.5
C16—S1—Ru1	114.42 (13)	S1—C15—H15C	109.5
C15—S1—Ru1	111.80 (14)	H15A—C15—H15C	109.5
O2—S2—C17	107.02 (17)	H15B—C15—H15C	109.5
O2—S2—C18	105.76 (17)	C13—C14—C9	120.6 (4)
C17—S2—C18	99.59 (19)	C13—C14—H14	119.7
O2—S2—Ru1	116.22 (12)	C9—C14—H14	119.7
C17—S2—Ru1	115.64 (13)	S1—C16—H16A	109.5
C18—S2—Ru1	110.93 (13)	S1—C16—H16B	109.5
C1—C2—C3	119.5 (4)	H16A—C16—H16B	109.5
C1—C2—H2	120.2	S1—C16—H16C	109.5
C3—C2—H2	120.2	H16A—C16—H16C	109.5
N3—N2—C6	110.8 (3)	H16B—C16—H16C	109.5
N3—N2—Ru1	134.0 (2)	S2—C17—H17A	109.5
C6—N2—Ru1	115.0 (2)	S2—C17—H17B	109.5
C1—N1—C5	117.9 (3)	H17A—C17—H17B	109.5
C1—N1—Ru1	126.7 (3)	S2—C17—H17C	109.5
C5—N1—Ru1	115.1 (2)	H17A—C17—H17C	109.5
C7—N4—N3	111.9 (3)	H17B—C17—H17C	109.5
C7—N4—C8	127.9 (3)	S2—C18—H18A	109.5
N3—N4—C8	120.2 (3)	S2—C18—H18B	109.5
C3—C4—C5	118.5 (4)	H18A—C18—H18B	109.5
C3—C4—H4	120.7	S2—C18—H18C	109.5
C5—C4—H4	120.7	H18A—C18—H18C	109.5
N2—C6—C7	107.1 (3)	H18B—C18—H18C	109.5
			
N1—C1—C2—C3	−0.7 (6)	C4—C5—C6—N2	−178.9 (3)
C4—C3—C2—C1	1.1 (6)	N1—C5—C6—C7	177.5 (4)
N4—N3—N2—C6	−0.2 (4)	C4—C5—C6—C7	−0.5 (6)
N4—N3—N2—Ru1	174.8 (2)	N3—N4—C7—C6	−0.3 (4)
C2—C1—N1—C5	−0.8 (6)	C8—N4—C7—C6	177.8 (3)
C2—C1—N1—Ru1	−174.9 (3)	N2—C6—C7—N4	0.2 (4)
C4—C5—N1—C1	1.9 (5)	C5—C6—C7—N4	−178.2 (4)
C6—C5—N1—C1	−176.1 (3)	C7—N4—C8—C9	−53.0 (5)
C4—C5—N1—Ru1	176.7 (3)	N3—N4—C8—C9	125.0 (3)
C6—C5—N1—Ru1	−1.3 (4)	N4—C8—C9—C14	−34.4 (5)
N2—N3—N4—C7	0.3 (4)	N4—C8—C9—C10	147.0 (3)
N2—N3—N4—C8	−178.0 (3)	C14—C9—C10—C11	−0.3 (5)
C2—C3—C4—C5	0.0 (5)	C8—C9—C10—C11	178.3 (3)
N1—C5—C4—C3	−1.5 (5)	C9—C10—C11—C12	1.2 (6)
C6—C5—C4—C3	176.3 (3)	C10—C11—C12—C13	−0.7 (6)
N3—N2—C6—C7	0.0 (4)	C14—C13—C12—C11	−0.7 (6)
Ru1—N2—C6—C7	−176.1 (2)	C12—C13—C14—C9	1.6 (6)
N3—N2—C6—C5	178.7 (3)	C10—C9—C14—C13	−1.1 (5)
Ru1—N2—C6—C5	2.7 (4)	C8—C9—C14—C13	−179.6 (4)
N1—C5—C6—N2	−0.9 (5)		

### (OC-6-32)-[1-Benzyl-4-(pyridin-2-yl-κN)-1H-1,2,3-triazole-κN3]dichloridobis(dimethyl sulfoxide-κS)ruthenium(II) (3) Hydrogen-bond geometry (Å, º)

**Table:**

*D*—H···*A*	*D*—H	H···*A*	*D*···*A*	*D*—H···*A*
C7—H7···O1^i^	0.95	2.11	3.031 (4)	164

Symmetry code: (i) *x*+1, *y*, *z*.
