# Supplementary figures and images for: Two isomers of [1-benzyl-4-(pyridin-2-yl-κN)-1H-1,2,3-triazole-κN 3]di­chlorido­bis­(dimethyl sulfoxide-κS)ruthenium(II)
Source: Acta Crystallogr E Crystallogr Commun. 2019 Jul 4;75(Pt 8):1108–11. doi: 10.1107/S2056989019008375 (PMC6690446; doi:10.1107/S2056989019008375)

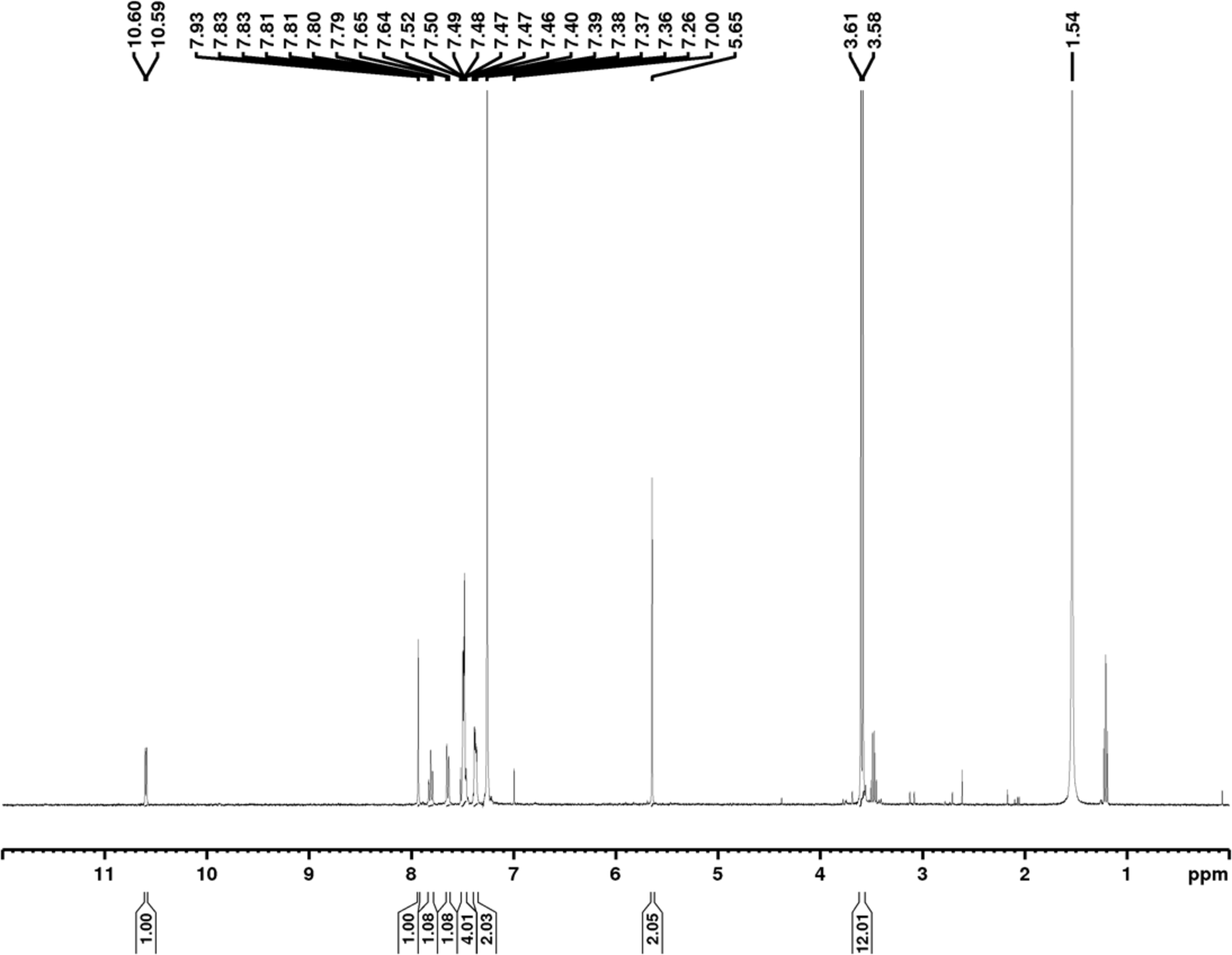

Supplement: Supplementary file 6 [file e-75-01108-sup6.tif]

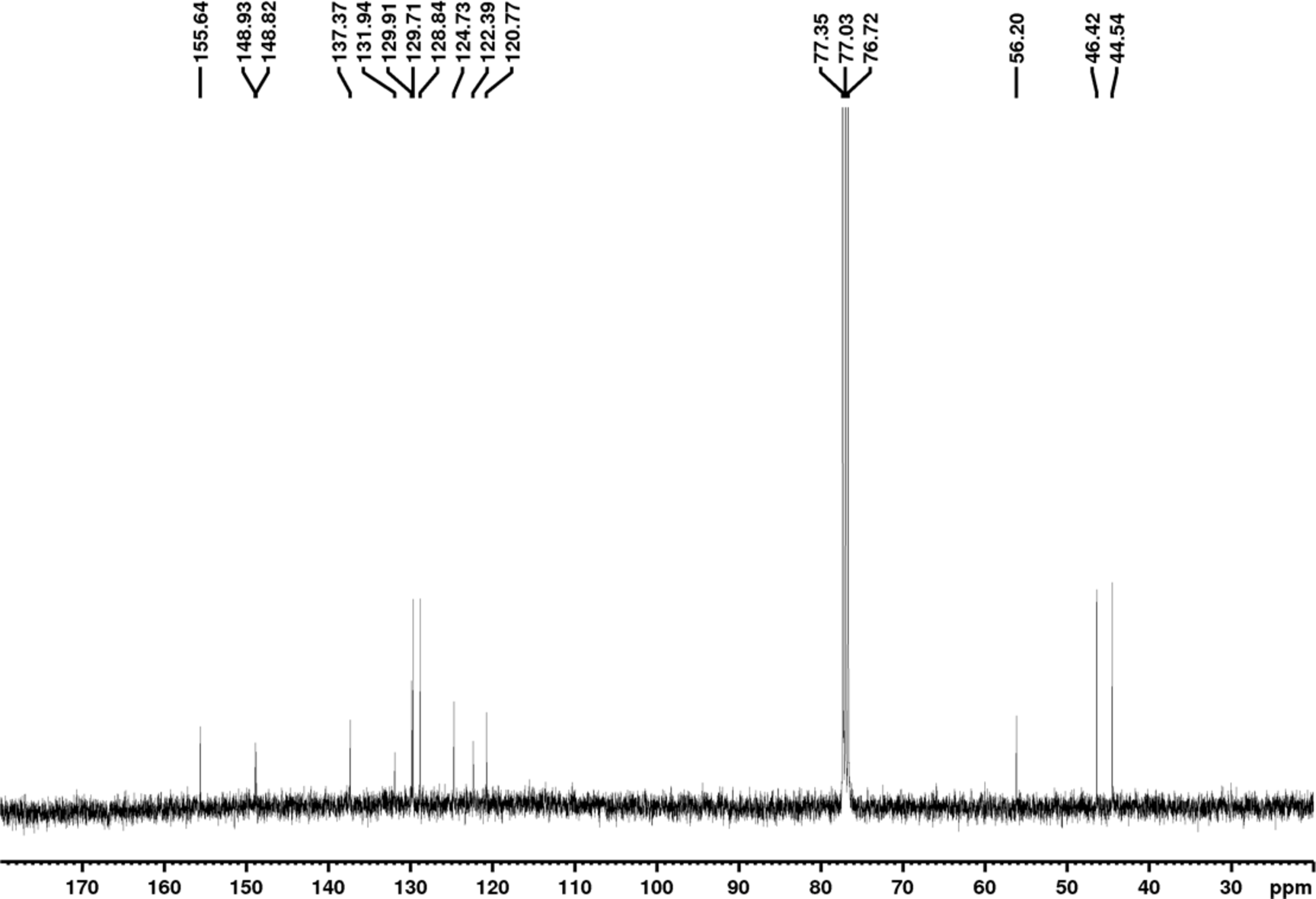

Supplement: Supplementary file 7 [file e-75-01108-sup7.tif]

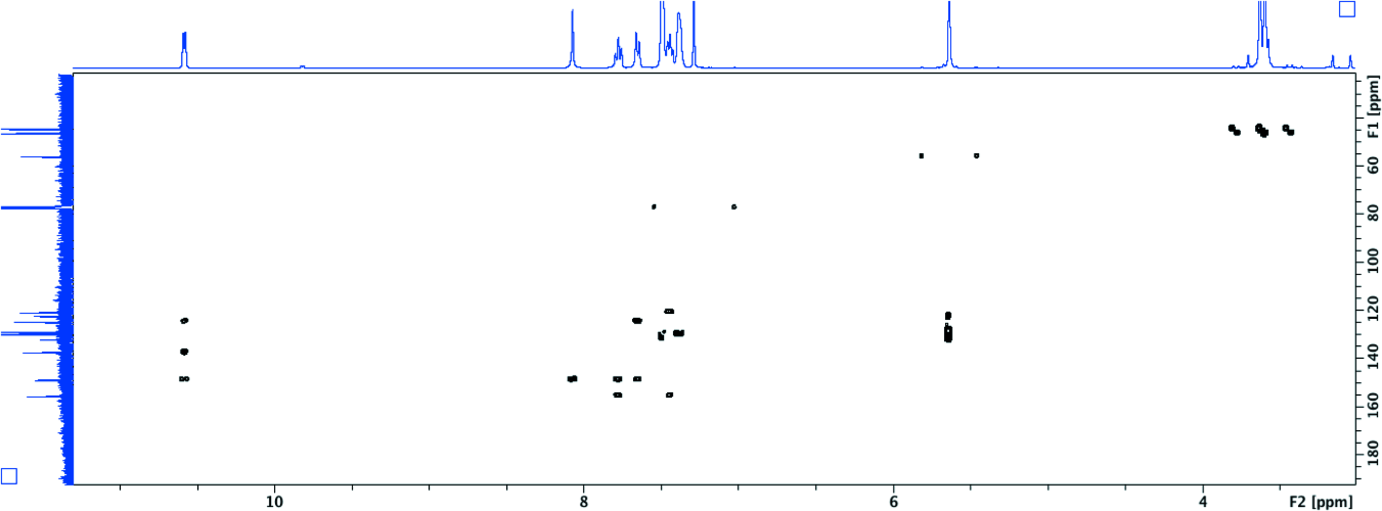

Supplement: Supplementary file 8 [file e-75-01108-sup8.tif]

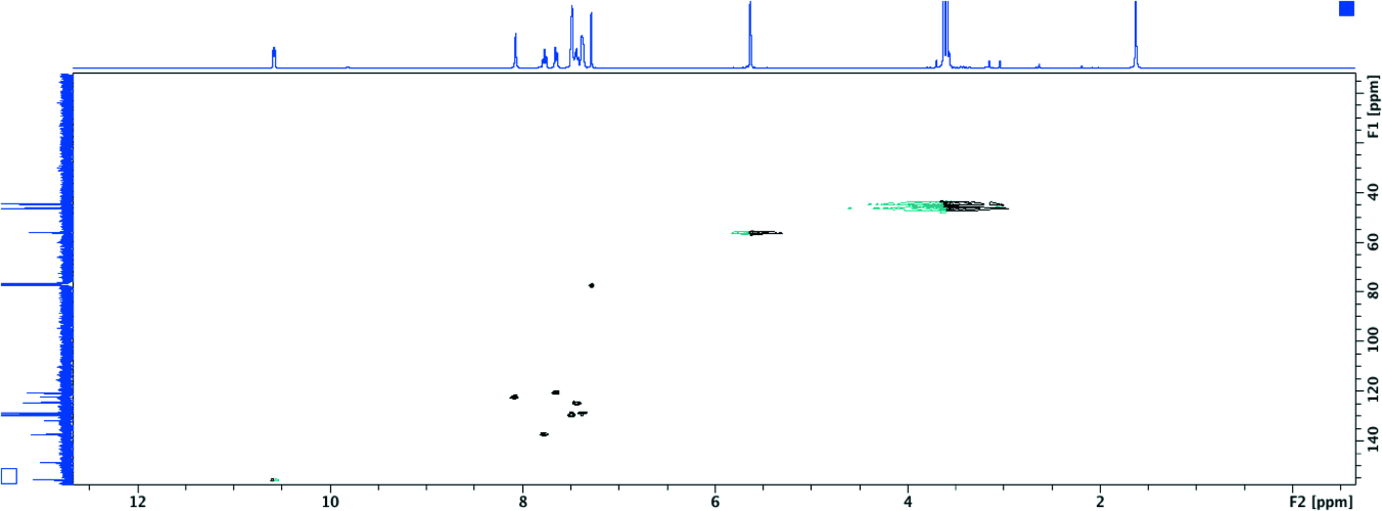

Supplement: Supplementary file 9 [file e-75-01108-sup9.tif]

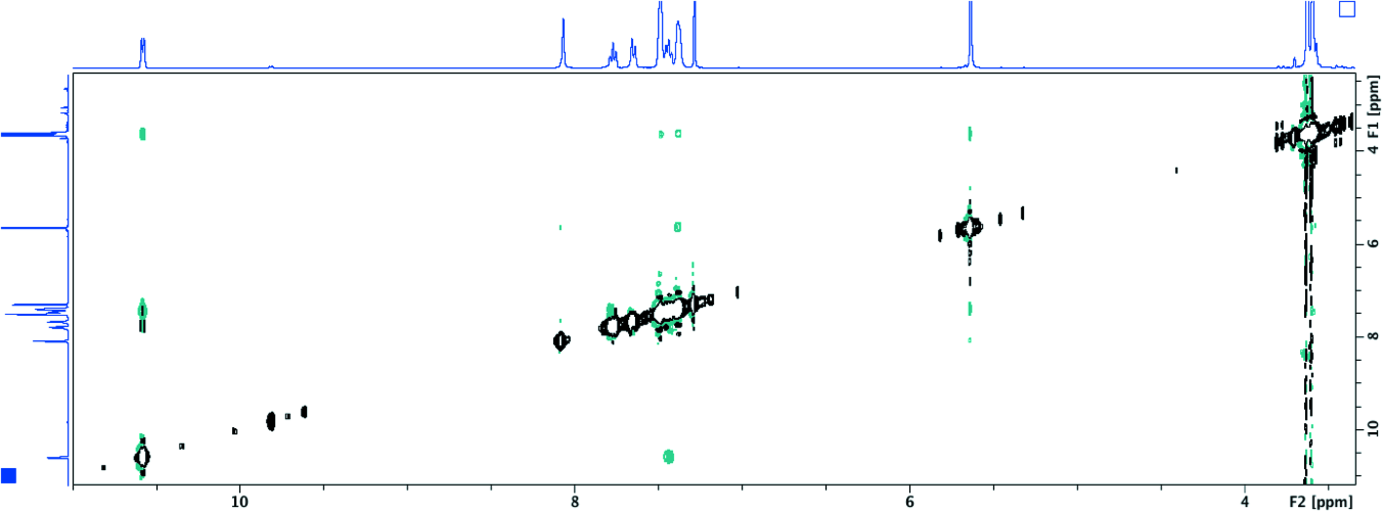

Supplement: Supplementary file 10 [file e-75-01108-sup10.tif]

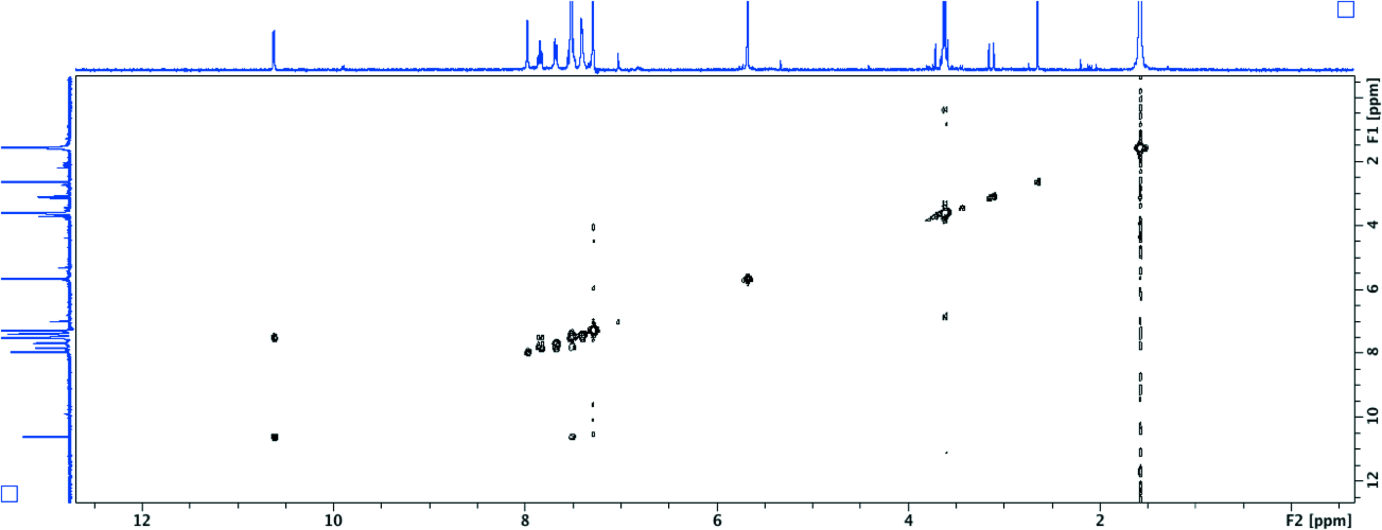

Supplement: Supplementary file 11 [file e-75-01108-sup11.tif]

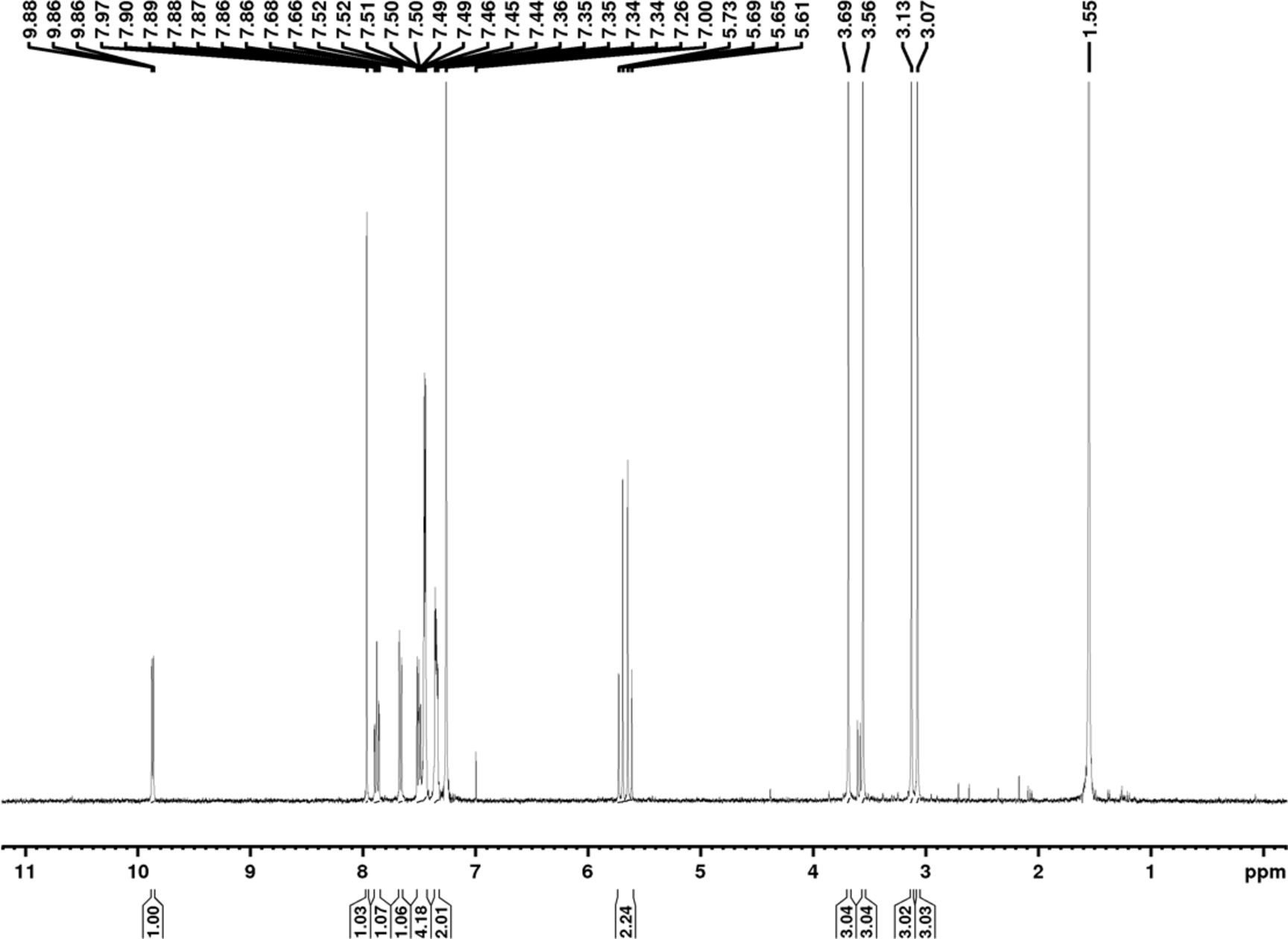

Supplement: Supplementary file 12 [file e-75-01108-sup12.tif]

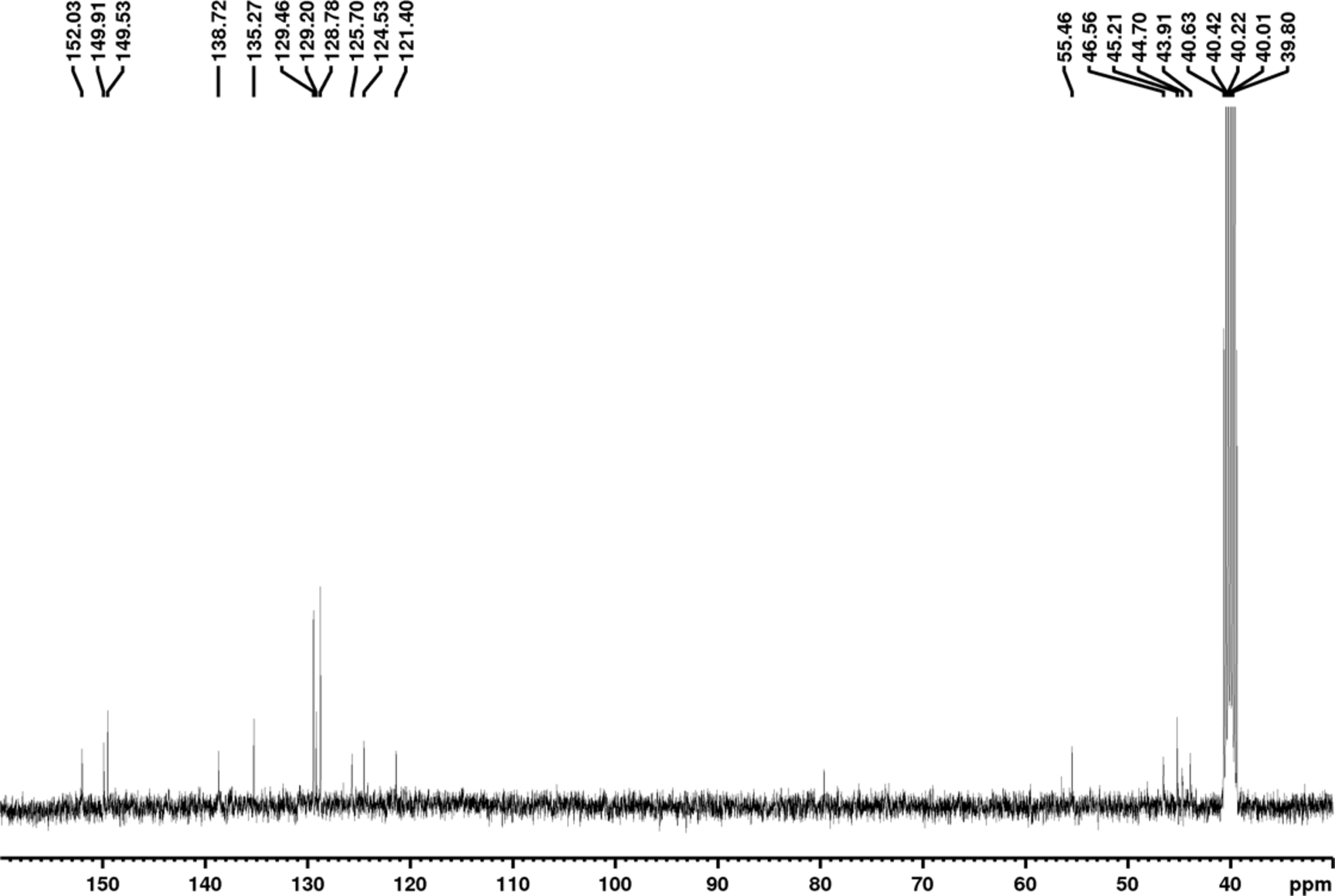

Supplement: Supplementary file 13 [file e-75-01108-sup13.tif]
